# Anatomo-surgical correlations in larynx cancer

**Published:** 2012-06-18

**Authors:** ALA Oancea, CR Popescu, P Bordei

**Affiliations:** * ENT&HNS Department, “Colţea” Clinical Hospital, Bucharest, Romania, “Carol Davila” University of Medicine and Pharmacy, Bucharest, Romania; ** Department of Anatomy, “Ovidius” University Constanta, Faculty of General Medicine, Romania

**Keywords:** larynx, partial laryngectomy, larynx anatomy

## Abstract

**Objective:** Cancer remains a crucial problem of contemporary medicine and the principle is perfectly true regarding the laryngeal cancer. Laryngologists are constantly searching for laryngeal cancer to provide functional and oncological surgical techniques. Conservative surgery in laryngeal cancer tries to keep enough laryngeal lumen and maintain the main laryngeal functions: breath, phonation, swallowing, by using open surgical techniques.

**Methods:** 412 patients with larynx neoplasms were operated by open surgical techniques between 1.01.2006 and 31.12.2008 in the ENT Clinic of “Colţea” Clinical Hospital. We selected 21 cases divided into three groups, each with 7 patients, to whom we have changed the type of surgery based on anatomical data. Careful preoperative selection is of outmost importance for the clinical outcome of the patients. Postoperative follow up was between 3 and 5 years.

**Results:** All the patients are alive, decannulated; they feed orally and are fully socially integrated.

**Conclusions:** In the light of anatomical structures analysis, indications of partial laryngeal surgery may be extended from where it is now, to accepting only total laryngectomy.

## Introduction

Recent research completed the elements of classic laryngeal anatomy by further studies using revolutionary techniques, offering new possibilities for assessing tumor extension and distribution of topographic lymphatic network, arguing the option of total or partial interventions.

The results of these researches have resulted in anatomical arguments that led to the interventional study conducted in the ENT Clinic of “Colţea” Clinical Hospital, between January 2006 and December 2008. 

## Method

21 patients to whom we have changed the classic type of surgery were selected based on anatomical data. These patients formed 3 groups: ***group I***, made up of 7 patients who underwent a cordectomy and a bilateral functional neck dissection; ***group II***, made up of 7 patients who underwent a hemilaryngectomy with a bilateral functional neck dissection instead of a total laryngectomy; ***group III***, made up of 7 patients who underwent a horizontal supraglottic laryngectomy with bilateral functional neck dissection instead of a total laryngectomy.

### Anatomic arguments:

1. Lymphatics of vocal folds – the free edge: rare, exist and they cross;

2. Paraglottic space: anterior - thyroepiglottic ligament; lateral – laminae of thyroid cartilage; posterolateral - pyriform sinus; posteromedian - aryepiglottic fold; medial - ventricular band and ventricle of Morgagni; superior in relation to preepiglottic space; inferior – elastic cone;

3. Preepiglottic space: anterior - thyrohyoid membrane; superior - thyroepiglottic ligament and vallecula; posterior - anterior face of epiglottis; inferior - epiglottis petiole.

## Results and Discussion

**Group I.** Starting from the first anatomic argument regarding the presence of lymphatic networks at vocal folds level, between 2006-2008, a number of 7 patients diagnosed with cancer of the median part of vocal cord was selected, without involving the anterior commissure and vocal apophysis, stage T1a, which we have performed according to the cordectomy and bilateral functional neck dissection. 

The fact that evidence of micrometastasis is a significant prediction factor in terms of tumor recurrence, proved after numerous trials, constituted an anatomic argument that led to the association of lymphadenectomy and cordectomy, even in cases of glottic tumor stage T1a.

These recently published studies have drawn attention to the high number of micrometastasis at the nodules level. Clinical definition of metastasis at the cervical lymphatic nodules is given by the presence of metastases that cannot be detected in routine microscopic examination with haematoxylin eosin coloration, but which can then be identified by histopathological techniques which are more sensitive [**1,2**]. Prognostic significance of the micrometastasis in cancer of the upper aero-digestive tract is not fully clarified. Studies in esophageal cancer have shown that evidence of micrometastasis represent a significant prediction factor in terms of tumor recurrence. Also, at the routine histopathological examination, patients (N-), but also (N+) at the specific examinations by immunohistochemistry, had a similar trend found in patients (N+) in the nodular histopathological examinations using classical methodology. Micrometastasis is more frequent than it is shown by current histopathological examination with haematoxylin-eosin coloration.

43 cordectomies were performed in “Colţea” Clinical Hospital, ENT department, between 2006-2008, of which we selected 7 cases that we have made functional bilateral neck dissection on, even though Treatment Guidelines recommendations are limited to the indication of cordectomy. All the 7 patients selected were diagnosed with cancer of vocal folds located in the median part, with mobile vocal fold, without involving the anterior commissure and vocal apophysis, qualifying for stage T1a of the disease. 

All the surgical interventions for the 7 selected cases were preceded by a tracheotomy. After a cordectomy through median thyrotomy, the operator piece was sent to histopathological examination in order to reconfirm the exact diagnosis and the assessment of safety margins. This was followed by an approach to the lodges of the major vessels with their opening and realization of functional bilateral neck dissection. The lymphatic nodules removed were sent to an immunohistochemical examination, specifying each nodule station. The tracheotomy performed to all the patients, for safety, was removed after 24 hours. 

Following the immunohistochemical examination, two of the patients presented micrometastasis at the laterocervical lymphatic nodules, which brought to discussion the need for a lymphadenectomy, also in patients who had no detectable clinical and paraclinical nodular invasion. In the case of these two patients, we had a modification of TNM classification from T1aNoMo to T1aN1Mo, which led to a change in the stage, from stage I to stage III of the disease, with a direct implication regarding therapeutic indications and a dramatic decrease of the prognostic. These two patients were enclosed in one new postoperative program following a course of adjuvant postoperative radiation and, until now, no tumor relapse was present. All 7 patients, including two patients with laterocervical micrometastasis, who performed postoperative radiotherapy, are alive, talk, feed orally, showed no tumor recurrence so far and are perfectly integrated socio-professionally.

**Group II.** The second anatomic argument result from the anatomical research refers to the anatomical description of the paraglottic space, whereas, at the oncologic level, this space changes the classic conception of the three glottic floors of the larynx and raise questions regarding the classic laryngeal cancer surgery guidelines. 

Paraglottic space was described as being a result of some studies using radioisotopes and immunofluorescence. The immediate result of these studies is the classic correlation between different localizations of neoplazic processes and their extension. Normally, this space contains fat, blood and lymphatic vessels. The anatomic paraglottic space is delimited anteriorly by the thyroepiglottic ligament, laterally by laminae of thyroid cartilage, posterolaterally by the pyriform sinus, posteromedially by the aryepiglottic fold, medially by the ventricular band and ventricle of Morgagni, superiorly it is in relation with the preepiglottic space, and inferiorly with the elastic cone, that presents a weak barrier for neoplazic processes, that can be extended unimpeded [**3**]. 

Theoretically, the relation to preepiglottic space is represented superiorly by a fibroelastic membrane, which consists of fibers of collagen and a small percentage of elastic fibers, resistant to neoplazic extension. There are authors who consider the preepiglottic spaces and the paraglottic space as being one periepiglottic space, apparently the name mismatch exists in order to underline the concrete anatomic data. This space is not reachable to classic laryngoscope and/or endoscopic examination. The examination of this space is possible only from an imagistic point of view, potentially accessible to radiologists specializing in larynx (ORL). Only CT or MRI can provide clues on the extent of tumor invasion by any of these areas, with major repercussions on the decision to total or partial larynx surgery [**4**]. 

16 hemilaryngectomy procedures were performed in “Coltea” Clinical Hospital, ENT department, between 2006 and 2008. Based on anatomical arguments described above, the CT images and endoscopic examination we changed the type of surgery in 7 patients with hemilaryngectomy, due to the patients for whom total laryngectomy indication represented a classic option. All the 7 patients presented an intrinsic tumor of the hemilarynx extended to three floors, with hipomobility of tumoral larynx, which has also caught the paraglottic space.

Each patient was verified endoscopically and by computer tomography for indemnity of arytenoid on the tumoral hemilarynx, one of the reasons for the practice of hemilaryngectomy was to allow patients to feed orally after the surgery. Four patients were in stage T3N0M0 and three in stage T3N1M0 [**5**]. Selected patients were aged less than 60 years. 

All the surgeries for the 7 selected cases were preceded by a tracheotomy. Hemilaryngectomy was performed with the preservation of the superior laryngeal nerve and of the arytenoid, the latter being free. Therefore, a tumor resection was done with the preservation of the swallowing, phonation and breathing. The intervention was completed with the bilateral functional neck dissection.

All the seven patients were decannulated postiradiation, keeping the safety cannula during radiotherapy. Currently, all the patients are alive, feed orally, speak, had no tumor recurrence, and are perfectly integrated socio-professionally. Regular ENT postoperative checks showed no evidence of local or regional recurrence.

**Fig. 1:**
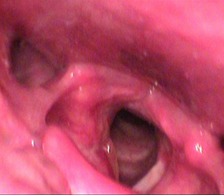
Laryngeal endoscopy: right hemilarynx tumor

**Fig. 2:**
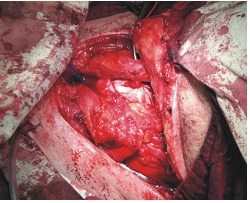
Hemilaryngectomy – surgical aspect

**Group III.** The third anatomic argument refers to the hyothyroepiglottic space. This is a celluloadipose tissue made up of two preepiglottic spaces in the shape of upper-based triangles. It consists of the space between the hyoid bone, the thyrohyoid membrane, the upper part of the thyroid cartilage situated anteriorly and the anterior face of the epiglottis situated posteriorly. A three-wall base and a bottom form each preepiglottic space (Boyer’s space). These spaces are of great interest for the oncologic surgery, because their achievement and integrity, conditions the surgery techniques used for malignant tumors of the laryngeal vestibular edges of the base of the tongue and larynx. The anterior wall of the space is made up of the hyoid bone from the top to the bottom, thyrohyoid membrane and the superior part of the thyroid cartilage, above the insertion of the epiglottis. The anterosuperior laryngeal artery enters into the preepiglottic space, perforating the thyrohyoid membrane 1 cm in front of the superior horn base of the thyroid cartilage. Two veins accompany it. The superior laryngeal nerve perforates the membrane in a plan that is deeper and upper than the vein, but it does not enter the space. The preepiglottic space is anterior to the muscular and aponeurotic layer of the neck and the subcutaneous layer through the hyoid bone, the thyrohyoid membrane and the thyroid cartilage. Between the thyrohyoid membrane and the deep cervical aponeurosis there is the serous bursa called the Boyer’s space, an inconstant cellular sliding layer with the superior laryngeal artery and superior branch of the superior laryngeal nerve inside. The posterior preepiglottic space corresponds to the laryngeal vestibule, especially with the anterior part of the ventricular band and the Morgagni's ventricle, throughout the epiglottis. Laterally and downwards, it corresponds to the anterior angle of the pyriform sinus. The preepiglottic space views the anterior commissure from which it is separated by the thyroepiglottic ligament, through the space bottom. It is in relation with the anterior part of the thyroarytenoid muscles and ligaments, including Boyer’s ligament. The roof of the preepiglottic space corresponds to the anterior part of the tongue base and tonsil. The latter’s height might hide the anterior recess. Its posterior space corresponds to the glossal face of the epiglottis. Laterally, it comes in relation with the lateral glossoepiglottic, pharyngoepiglottic fold and the aryepiglottic fold. The roof of the preepiglottic space represents a vallecula in terms of endoscopy. Medially, it is in relation with the preepiglottic controlateral homologous space through the intermediation of the preepiglottic membrane. The extension of the tumor process into the hyothyroepiglottic space is observed through an imagistic point of view, by highlighting the tumoral tissue that replaces the normal fat space level. The surgery indication is determined by the quantification of the degree of tumor invasion of concern [**6**]. 

The third change of the classic category of surgical intervention, based on anatomical data mentioned above, was the total laryngectomy for those patients whose hyothyroepiglottic space was invaded with horizontal supraglottic laryngectomy, with resection of tongue base.

38 horizontal supraglottic laryngectomies were made in “Colţea” Clinical Hospital, ENT Department, in the period 2006-2008, out of which, seven cases were selected and based on anatomical data, we changed the indication for surgery from total laryngectomy in horizontal supraglottic with the resection of the base of tongue. The rationale of this approach met the anatomical conditions, preoperative endoscopic images, CT images and, it should be noted that these seven patients were selected according to the criteria set out, in which the argument age and mental condition were prevailing. All the seven patients had a tumor located in the anterior commissure, extended to the laryngeal face of the epiglottis and on the base of tongue, preserving the vocal cord mobility. The cartilage integrity of each patient was tested clinically, intraoperatively and from an imagistic point of view. Three patients were in stage T4aN1M0 and four in stage T4aN2M0 [**5**].

All the surgeries for the 7 selected cases were preceded by tracheotomy. Horizontal supraglottic laryngectomy was performed with the resection of the base of tongue, Leroux-Berger technique and bilateral functional neck dissection.

All the seven patients were decannulated postiradiation, keeping the safety cannula during radiotherapy [**7-10**]. Currently, all the patients are alive, feed orally, speak, had no tumor recurrence, and are perfectly integrated socio-professionally. Regular ENT postoperative checks showed no evidence of local or regional recurrence.

**Fig. 3,4:**
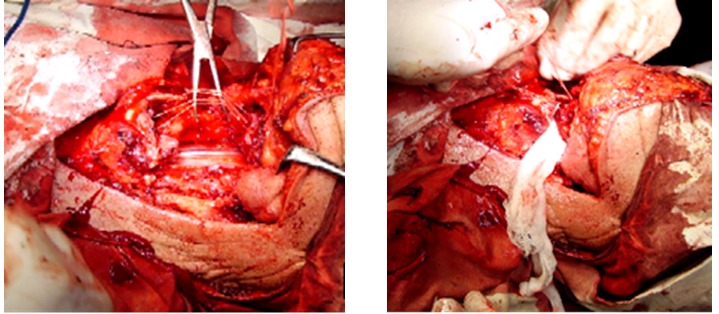
Horizontal Supraglottic Laryngectomy with resection of tongue; basic aspects of surgical intervention

## Conclusions

The radical curative treatment of the neoplasm of larynx T + N addresses and is the fundamental principle of Oncology, based on correct staging and exact safety cuffs obtained for T and lymphadenectomy harmless.

The choice of therapeutic methods, including the type of surgical intervention is particularly important, based on histological or immunohistochemical confirmation of malignant nature of the tumor set out clearly and communicated to patients in the pre-therapeutic discussion. 

The paper brings to light the recommendations of Treatment Guidelines 2011, following the positive effects of surgical interventions involving changes in TNM's and therefore the staging of larynx carcinoma, with direct implications on prognosis.
